# Electroencephalographic and Behavioral Effects of Intranasal Administration of a Na^+^, K^+^-ATPase-Activating Antibody after Status Epilepticus

**DOI:** 10.1021/acschemneuro.4c00141

**Published:** 2024-07-11

**Authors:** Fernanda Kulinski Mello, Tuane Bazanella Sampaio, Bruna Neuberger, Michele Pereira Mallmann, Michele Rechia Fighera, Luiz Fernando Freire Royes, Ana Flávia Furian, James W. Larrick, Mauro Schneider Oliveira

**Affiliations:** † Graduate Program in Pharmacology, Federal University of Santa Maria, Santa Maria 97105-900, Brazil; ‡ Department of Neuropsychiatry, Federal University of Santa Maria, Santa Maria 97105-900, Brazil; § Department of Sports Methods and Techniques, Federal University of Santa Maria, Santa Maria 97105-900, Brazil; ∥ Panorama Research Institute, 1230 Bordeaux Dr., Sunnyvale, California 94089, United States

**Keywords:** Epilepsy, neuroprotection, pilocarpine, EEG, nose-to-brain delivery, DRRSAb

## Abstract

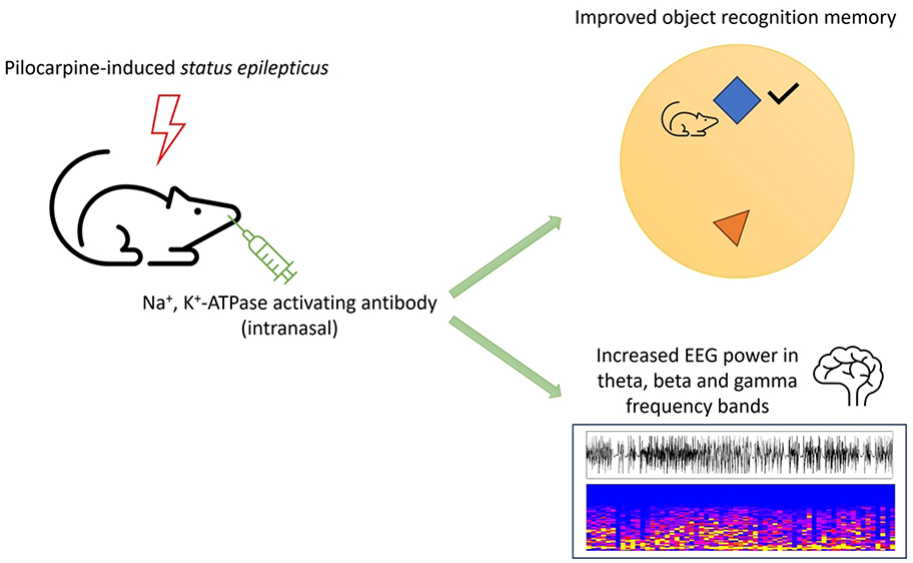

Status epilepticus (SE) is a medical emergency associated with high mortality and morbidity. Na^+^, K^+^-ATPase, is a promising therapeutic target for SE, given its critical role in regulation of neuron excitability and cellular homeostasis. We investigated the effects of a Na^+^, K^+^-ATPase-activating antibody (DRRSAb) on short-term electrophysiological and behavioral consequences of pilocarpine-induced SE. Rats were submitted to pilocarpine-induced SE, followed by intranasal administration (2 μg/nostril). The antibody increased EEG activity following SE, namely, EEG power in theta, beta, and gamma frequency bands, assessed by quantitative analysis of EEG power spectra. One week later, DRRSAb-treated animals displayed less behavioral hyperreactivity in pick-up tests and better performance in novel object recognition tests, indicating that the intranasal administration of this Na^+^, K^+^-ATPase activator immediately after SE improves behavioral outcomes at a later time point. These results suggest that Na^+^, K^+^-ATPase activation warrants further investigation as an adjunctive therapeutic strategy for SE.

## 1 Introduction

Epilepsy is a chronic neurological disease that affects over 65 million people worldwide.^1^ The disease is characterized by a predisposition to generate recurrent and spontaneous seizures, which present variable duration and with a broad range of signs and symptoms.^2^ When a seizure lasts more than 5 min, it is defined as *status epilepticus* (SE).^3^ SE is the most severe seizure disorder. SE is often life-threatening and can lead to epilepsy.^3^ Apart from the seizures, patients and their families with epilepsy also suffer with medical, psychiatric and cognitive consequences, such as depression and memory impairment.^4^

Na^+^, K^+^-ATPase is a ubiquitous enzyme responsible for the transport of three Na^+^ ions out and two K^+^ ions into cells, with the energy provided by ATP.^5^ This movement creates an electrochemical gradient across the membrane that is critical for homeostasis and function of all mammalian cells.^5^ In the brain, Na^+^, K^+^-ATPase is the major regulator of neuron excitability and its activity directly affects neural network excitability.^6^ In this context, decreased Na^+^, K^+^-ATPase activity is postulated to be a major factor in seizures and epilepsy and suggests this enzyme to be a potential new target for the treatment of epilepsy. Regarding this point, previous studies have shown that a Na^+^, K^+^-ATPase activating antibody, DRRSAb, restores glutamate release and glucose uptake to normal levels in hippocampal slices from epileptic mice.^7^ Moreover, the intrahippocampal injection of DRRSAb decreases seizure susceptibility in mice 2 months after SE.^8,9^ However, the short-term effects of DRRSAb on SE have never been tested.

Numerous studies support nose-to-brain delivery as a noninvasive route to transport a drug directly into the brain. This delivery method is a more comfortable route, relatively safe and fast, and the systemic effects of a drug are minimized.^10,11^ Nasal administration constitutes a potential strategy to improve treatment for several neurologic conditions.^12^ Commercial intranasal preparations contain Sumatriptan or zolmitriptan for migraine, (*S*)-ketamine for depression, and naloxone for opioid overdose.^12^ Moreover, nasal sprays containing a benzodiazepine like diazepam or midazolam have been available as rescue therapy for acute repetitive seizures (i.e., seizure clusters).^12^

Thus, based on the premise that Na^+^, K^+^-ATPase is a target for seizure control, and that the intranasal route may offer advantages in epilepsy treatment, we aimed to investigate the effects of intranasally administered DRRSAb on electroencephalographic and behavioral parameters after pilocarpine-induced SE in rats. An increasing number of studies have used the intranasal route to deliver therapeutic antibodies to the brain and eventually achieve beneficial effects.^13,14^ Nevertheless, to the best of our knowledge, this is the first report showing the use of an intranasally administered antibody in the context of *status epilepticus.*

## 2 Results and Discussion

**Figure 1:**
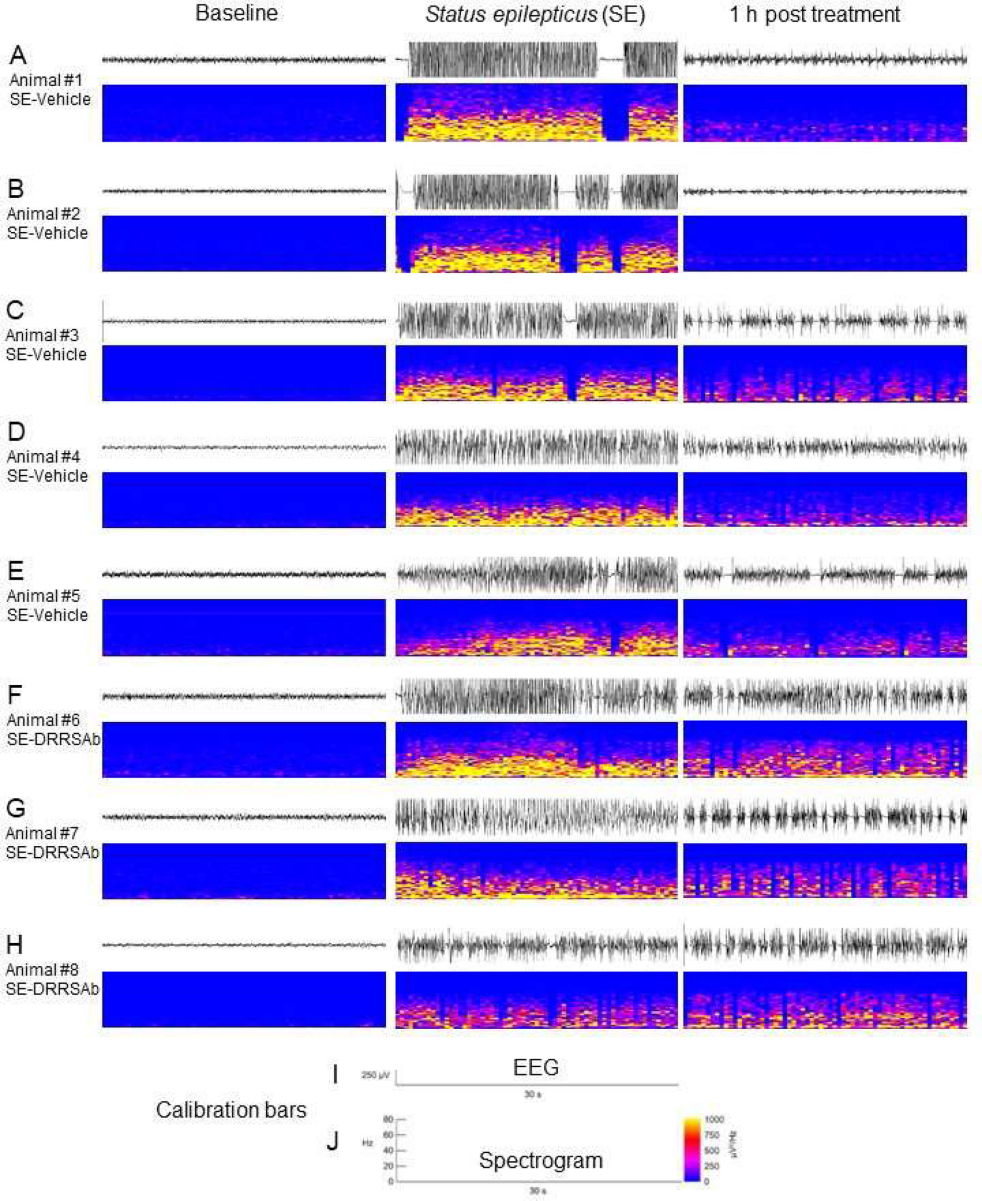
EEG recording and power spectra analyses on baseline (left), during SE (middle), and 1 h after treatment (right) with diazepam plus vehicle (A–E) or diazepam plus Na+, K+-ATPase activating antibody DRRSAb (F–H). Calibration bars for EEG and spectrogram are shown in panels (I) and (J).

Pilocarpine induced a typical limbic SE in all animals submitted to the procedure. Animals were treated with the Na^+^, K^+^-ATPase activating antibody 1 h after SE, and EEG was recorded for one more hour. Representative recordings from each animal after vehicle or DRRSAb administration are shown in Figure 1. At this time point, recordings showed typical epileptiform patterns, which were characterized by continuous spiking and large amplitude irregular activity. Interestingly, 1 h after intranasal administration, EEG of animals submitted to SE and treated with diazepam plus vehicle (Figure 1A–E) presented attenuated epileptiform activity and EEG power when compared to EEG from animals submitted to SE and treated with diazepam plus Na^+^, K^+^-ATPase activating antibody DRRSAb (Figure 1F–H).

**Figure 2:**
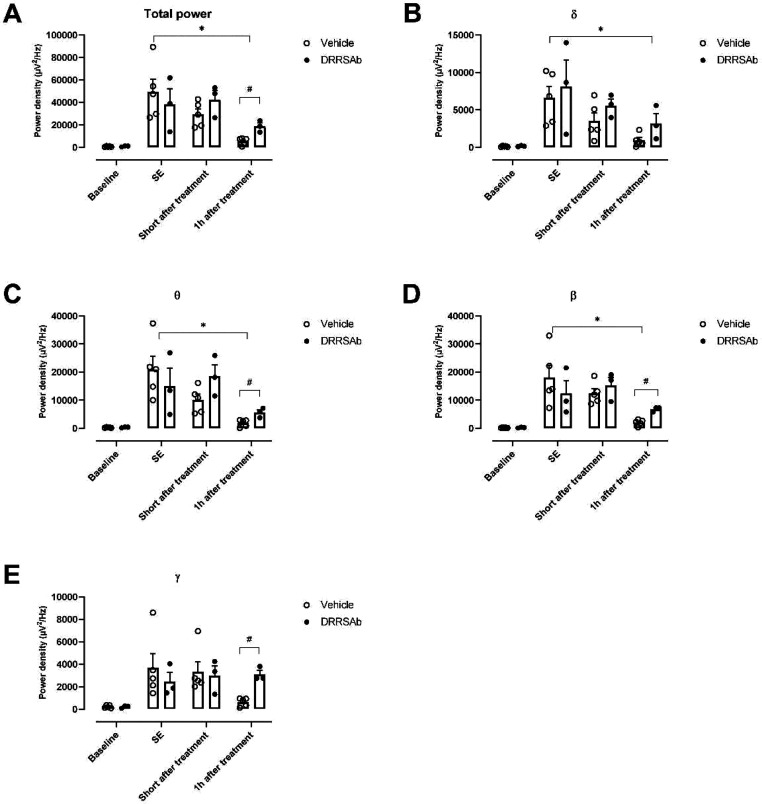
Time-course quantitative analysis of EEG power spectra of animals during the SE, a short time (5–10 min) after treatment with diazepam plus vehicle or diazepam plus DRRSAb, and 1 h after treatment. (A) EEG total power, EEG power in (B) delta, (C) theta, (D) beta, and (E) gamma frequency bands. Data are expressed as mean + SEM of 3–5 animals/group. *Denotes *P* < 0.05 vs SE; ^#^denotes *P* < 0.05 vs vehicle-treated animals at the same time (two-way ANOVA followed by post hoc Tukey’s multiple comparisons test or Fisher’s least significant difference test).

Time-course quantitative analysis of EEG power spectra of animals submitted to SE is shown in Figure 2. EEG total power of animals submitted to SE and treated with diazepam and vehicle decreased at 60 min after drug administration [F (1.231, 7.383) = 7.927; P = 0.0212] (Figure 2A) when compared to SE. Interesting, a similar decreasing effect was not found in EEG total power of animals submitted to SE and treated with diazepam and DRRSAb (Figure 2A). Moreover, EEG total power increased in DRRSAb-treated animals 60 min after SE when compared with vehicle-treated controls [t(6) = 4.599; P = 0.0037] (Figure 2A).

To evaluate EEG changes elicited by intranasal injection of the Na^+^, K^+^-ATPase activator DRRSAb, we performed a quantitative analysis of the EEG recordings. We found that EEG power in delta [F (1.338, 8.028) = 6.994; P = 0.0239] (Figure 2B), theta [F (1.292, 7.751) = 9.223; P = 0.0134] (Figure 2C), and beta [F (1.211, 7.264) = 6.600; P = 0.0321] (Figure 2D) frequency bands decreased at 60 min after drug administration in EEG from animals submitted to SE and treated with diazepam and vehicle when compared to SE. No statistically significant changes were found in EEG power in delta, theta, beta, or gamma frequency bands at 60 min after drug administration of animals submitted to SE and treated with diazepam and DRRSAb. Importantly, post hoc analyses indicated that EEG power in theta (Figure 2C), beta (Figure 2D), and gamma (Figure 2E) frequency bands increased in DRRSAb-treated animals 60 min after SE when compared with vehicle-treated animals.

**Figure 3:**
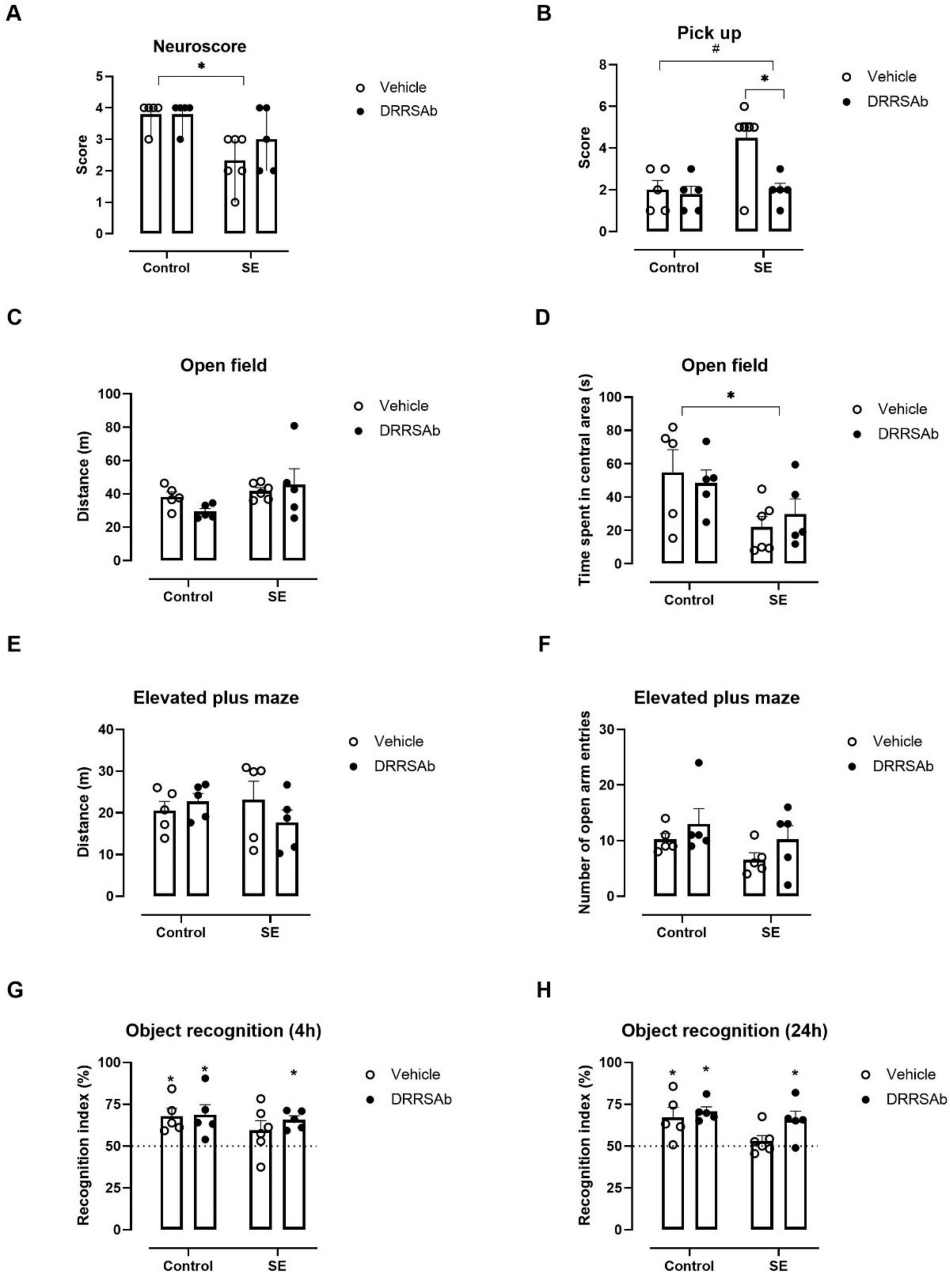
Behavioral parameters carried out from 7 days after SE induction and DRRSAb or vehicle treatments. (A) Neuroscore; *denotes a main effect of SE (*P* < 0.05; Scheirer-Ray-Rare nonparametric extension of two-way ANOVA), data are expressed as median + interquartile range. (B) Pick-up; *denotes *P* < 0.05 vs vehicle-treated animals, and ^#^*P* < 0.05 vs SE animals (two-way ANOVA, Tukey’s test). (C,D) Open field and (E,F) elevated plus maze; *denotes a main effect of SE (*P* < 0.05; two-way ANOVA, Tukey’s test). (G, H) Object recognition tests; *denotes *P* < 0.05 vs theorical value of 50% (one sample *t* test). Data are expressed as mean + SEM of 5–6 animals/group.

We also investigated whether EEG modulation by DRRSAb after SE would alter the behavioral impairments that often follow SE. To this purpose, animals were tested in a behavioral test battery from 7 days after SE (Figure 3). SE impaired neuromotor function, evaluated in the neuroscore [F (1,17) = 8.491; P = 0.0024] (Figure 3A) and pick-up [F (1,17) = 6.652; P = 0.0195] (Figure 3B) tests. In the neuroscore test, no effects of DRRSAb were detected. However, the Na^+^, K^+^-ATPase activating antibody improved behavioral reactivity scores in the pick-up test, indicating a beneficial effect (SE × DRRSAb interaction [F (1,17) = 4.827; P = 0.0422]) (Figure 3B). To evaluate locomotor/exploratory function and anxiety-like behavior, we performed open field and elevated plus maze tests. No changes were found in the total distance traveled in the open field (Figure 3C) or elevated plus maze (Figure 3E) apparatuses, suggesting no locomotor deficits caused both SE and DRRSAb treatment. On the other hand, SE decreased the time spent in the central area during the open field test ([F (1,17) = 7.693; P = 0.0130]) (Figure 3D) but did not alter the number of open arm entries in the elevated plus maze test (Figure 3F). For the statistical analysis of object recognition test data, we employed a One sample t test to verify whether some group would differ from the theorical values that would be expected if the animals explored the objects by chance (i.e., 50%). For instance, if animals do not remember the object used during the training session (old object), they would be expected to equally explore both objects. Thus, the one sample t test allows one to detect a true deficit in the animal’s performance during the test. Results revealed that vehicle-treated animals submitted to SE were not able to discriminate the novel object at 4 h (t(5) = 1.649; P = 0.16) (Figure 3G) or 24 h (t(5) = 0.974; P = 0.375) (Figure 3H) after training, suggesting impairment in the short- and long-term recognition memories. Importantly, animals submitted to SE that received intranasal injection of DRRSAb spent more time exploring the novel object at 4 h (t(4) = 6.429; P = 0.003) (Figure 3G) or 24 h (t(4) = 2.999; P = 0.04) (Figure 3H) after training, indicating that the Na^+^, K^+^-ATPase activating antibody prevented the SE-induced impairment on the novel object recognition ability.

In the present study, we showed that intranasal administration of the Na^+^, K^+^-ATPase activating antibody DRRSAb increased EEG activity following pilocarpine-induced SE. Moreover, quantitative analysis of EEG power spectra showed that DRRSAb increased EEG power in theta, beta, and gamma frequency bands. Importantly, DRRSAb-treated animals displayed less behavioral hyperreactivity in the pick-up test and better performance in the novel object recognition test, indicating that intranasal injection of a Na^+^, K^+^-ATPase activator after SE improves behavioral outcomes from 1 week later.

Na^+^, K^+^-ATPase is a major contributor of brain excitability and maintenance of the resting membrane potential. Accordingly, mutations in the enzyme as well as changes in its activity are associated with several neurologic diseases, including epilepsy.^5,6^ Moreover, many studies have established that ouabain, a classic Na^+^, K^+^-ATPase inhibitor, causes seizures and hyperexcitability.^15−17^ In addition, decreased Na^+^, K^+^-ATPase activity was found in human epileptic brain^18,19^ as well as in the hippocampus of rats^20^ and mice^21^ after pilocarpine-induced SE. Altogether, these data support the idea that a decrease of Na^+^, K^+^-ATPase activity plays a role in the initiation and/or maintenance of seizures. Regarding this point, our group has been investigating the hypothesis that DRRSAb, a Na^+^, K^+^-ATPase activator, may act as an anticonvulsant and/or neuroprotective drug.

The DRRSAb antibody binds to 897DVEDSYGQQWTYEQR911 (D-R) region of the Na^+^, K^+^-ATPase alpha subunit,^9^ causing a conformational change that enhances Mg^2+^/ATP affinity and increases Na^+^/K^+^ transport.^22^ Our group have shown that the hippocampal injection of DRRSAb delayed the onset of myoclonic seizures in mice challenged with PTZ 60 days after SE,^8^ and restored the locomotor activity in the open field test 60 days post SE.^7^ However, the effects of Na^+^, K^+^-ATPase activation in the period immediately after SE have not been investigated. This is an intriguing prospect as, Fernandes and colleagues (1996) demonstrated that Na^+^, K^+^-ATPase activity is significantly reduced in the rat hippocampus 60 min after SE.^20^

In the present study, we treated the animals with Na^+^, K^+^-ATPase activator DRRSAb immediately after SE, in an attempt to circumvent the SE-elicited decrease in pump activity.^20^ Our results indicate that intranasal administration of DRRSAb after SE elicited a protective effect assessed 1 week later. In fact, SE rats showed worse performance in the neuroscore, pick-up, open field, and object recognition tests compared to the controls, confirming the expected impairment by the SE model. Conversely, DRRSAb-treated rats displayed better performance in the pick-up and object recognition tests, demonstrating a protective effect of the Na+, K+-ATPase activation in the immediate period after SE. To some extent, these behavioral improvements agree with those of Zhao et al., who have shown that optogenetic stimulation of astrocytes attenuates kainic-acid-induced seizures in mice via activation of Na^+^, K^+^-ATPase. This anticonvulsant effect was independent of Ca^2+^ signaling, and instead is related to astrocytic Na^+^, K^+^-ATPase -mediated K^+^ buffering, which activity-dependently inhibits firing in highly active pyramidal neurons during seizures.^23^

The downstream mechanisms underlying our observed beneficial effects have not been investigated in the present study; we expect selected signaling pathways to play a key role. For example, DRRSAb-induced cardioprotection against ischemic injury in cardiomyocytes and isolated hearts was reversed by ERK1/2 or phosphoinositide 3-kinase (PI3K)/Akt inhibitors,^9^ suggesting activation of those pathways by the Na^+^, K^+^-ATPase activating antibody. Interestingly, the ERK pathway inhibitor SL327 worsens pilocarpine-induced seizures: animals treated with SL327 had higher seizure-related mortality than vehicle-treated animals did.^24^ In addition, Akt activation in the hippocampus correlates negatively with seizure occurrence after SE, since higher phosphorylated Akt levels were observed in periods when seizures are no longer seen.^25^ Therefore, the possibility that DRRSAb activates ERK and/or Akt after SE resulting in beneficial effects at later time points after SE is an interesting possibility to be addressed in future studies.

The changes in the power spectrum after intranasal injection of DRRSAb is remarkable. Epilepsy has been considered a paroxysmal cerebral dysrhythmia,^26^ and therefore, changes in the normal rhythms are useful biomarkers of a pathological neural network.^27^ Our present results showed that DRRSAb increased EEG power in theta, beta, and gamma frequency bands after SE. This profile of multiple changes seems compatible with the ubiquitous expression of Na^+^, K^+^-ATPase across different brain areas and cell types. The activation of survival signaling cascades by DRRSAb may be linked to the changes in EEG power spectra.

Regarding this point, Trevio and colleagues studied the beta-gamma oscillations in the CA3 area of the hippocampus in slices from rats after PTZ-induced seizures. They showed that these oscillations are modulated by aberrant GABA neurotransmission from the dentate gyrus (DG) and can generate inhibitory postsynaptic potentials in the pyramidal cells.^28^ Considering that generalized seizures are followed by a period of depression, during and after which memory and cognitive deficits develop,^28^ aberrant activity-dependent tonic inhibition may constitute a mechanism contributing to such deficits. This increased inhibition can probably protect against the additional generation of seizures^28^ but, on the other hand, could lead to behavioral and cognitive deficits.^28^ In this context, the DRRSAb-elicited increase of beta and gamma power in comparison to vehicle-treated SE animals may reflect a relief of the aberrant inhibition occurring in the immediate period after SE. This in turn would contribute to improved performance in behavioral tests after recovery of SE from 1 week later.

In summary, we showed that intranasal administration of the Na^+^, K^+^-ATPase activating antibody DRRSAb after pilocarpine-induced SE increased EEG power in theta, beta, and gamma frequency bands 1 h thereafter, and that these changes may be related to improved behavioral outcomes from 1 week later. More studies are necessary to understand the mechanisms by which those effects occur and the possible translational application of intranasal administration of Na^+^, K^+^-ATPase activating antibodies.

## 3 Materials and Methods

### 3.1 Animals

Thirty-two male Wistar rats, aged 30–40 days (70–150 g) were maintained in controlled conditions (temperature 22 ± 2 °C, 12 h light/dark cycle) and with free access to water and food (Purotrato, Santa Maria, RS, Brazil). All experiments were in accordance with national legislation (Brazilian Council of Animal Experimentation – CONCEA) and approved by Ethics Committee for Animal Research of the Federal University of Santa Maria (approval number 1879071019).

### 3.2 Drugs and Reagents

The Na^+^, K^+^-ATPase activating antibody, DRRSAb, was kindly provided by Dr. James W. Larrick (Panorama Research Inc., Sunnyvale, CA, USA) and diluted in a saline solution. Pilocarpine was obtained from Sigma-Aldrich (St. Louis, MO, USA) and dissolved in saline solution.

### 3.3 Experimental Design

Animals were implanted with epidural electrodes following the protocol described in detail elsewhere.^29^ After a 72-h recovery period, animals were subjected to pilocarpine-induced SE^29^ and intranasal treatment. To this purpose, animals were treated with DRRSAb 2 μg/nostril or vehicle (NaCl 0.9%) 5 μL/nostril. Immediately after diazepam treatment, a 7 mm PE-50 tube was insert in the animal’s nostrils, and 5 μL of DRRSAb or vehicle was intranasally administered at a speed of 10 μL/min, using a 10 μL microsyringe (Hamilton, USA) and an infusion pump (Insight, Brazil).

**Figure 4:**
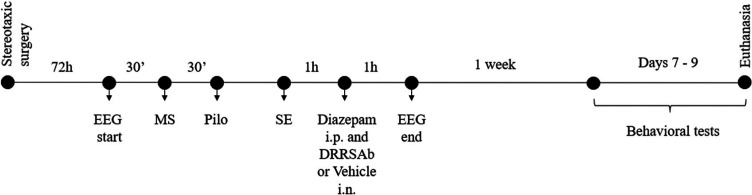
Experimental design. After 30 min of basal electroencephalographic recording (EEG), rats received methyl-scopolamine (MS) 1 mg/kg i.p., and 30 min thereafter a single injection of pilocarpine hydrochloride (Pilo) 300 mg/kg i.p. or vehicle (NaCl 0.9%; control group). One hour after the *status epilepticus* (SE) start, animals received diazepam (10 mg/kg i.p.) and DRRSAb 2 μg/nostril or vehicle (NaCl 0.9%; 5 μL/nostril), and the EEG was recorded until 1 h after. Behavioral tests were performed 7– 9 days after the pilocarpine protocol.

Animals were allocated into four groups; Group 1 (control-vehicle): NaCl 0.9% ip + NaCl 0.9% i.n. (5 μL/nostril); Group 2 (control-DRRSAb): NaCl 0.9% i.p. + DRRSAb 2 μg/nostril i.n.; Group 3 (SE-vehicle): pilocarpine 300 mg/kg i.p. + NaCl 0.9% i.n. (5 μL/nostril); Group 4 (SE-DRRSAb): pilocarpine 300 mg/kg i.p. + DRRSAb 2 μg/nostril i.n.. The timeline of experimental procedures is depicted in Figure 4.

In the present study, we lost 11 animals because of SE; 5 of them during SE itself and 6 during the week following SE. Such numbers are within the expected range according to previous work aimed to evaluate mortality in the pilocarpine model of status epilepticus.^30^

### 3.4 EEG Quantitative Analysis

EEG recordings of animals submitted to SE were analyzed off-line using standard functions of LabChart 7.2 software (AD Instruments) under the strategy described in detail elsewhere.^8^ Epochs containing artifacts were determined by a visual inspection of the EEG recordings and excluded from the analysis. One vehicle- and two DRRSAb-treated animals with artifact-prone EEG recordings were excluded from quantitative EEG analysis.

### 3.5 Behavioral Tests

Starting 7 days after the SE protocol, animals were assessed in a battery of behavioral tests. At day 7, animals were subjected to open field, neuroscore, and pick-up test^29^ to determine neuromotor performance, locomotion, and anxiety-like behavior. During the days 8 and 9, object recognition test,^31^ was carried out to evaluate the short-term and long-term memory. After the long-term memory test, the anxiety-like behavior was measured in the elevated plus maze test^32^ at day 9. The behavioral test battery was conducted always between the light phase of the circadian cycle. All behavioral tasks were scored by a participant that was blinded to treatments, and ANY Maze video Tracking system was used to extract data from the videos (Stoelting Co., Wood Dale, IL, USA).

### 3.6 Statistical Analysis

EEG and behavioral data were analyzed by two-way ANOVA followed by post hoc Tukey’s multiple comparisons test or Fisher’s least significant difference test when appropriate. Neuroscore data was analyzed by Scheirer-Ray-Rare nonparametric extension of two-way ANOVA. Object recognition test performance was evaluated by the One sample *t* test using 50% as the hypothetical value. Probability of *P* < 0.05 was considered statistically significant, all data are expressed as mean ± SEM, and all statistical analyses were done using GraphPad Prism 8.0 (GraphPad, San Diego, CA, USA).

## Data Availability

The data that support the findings of this study are available from the corresponding author upon reasonable request.
